# Single-cell transcriptomic insights into the intrinsic cardiac nervous system: diversity, development, and neuro-cardiac interactions

**DOI:** 10.3389/fgene.2026.1833404

**Published:** 2026-06-30

**Authors:** Jihao Zheng, Ruotong Wu, Chujiao Gu, Changsheng Chen, Linsheng Shi

**Affiliations:** 1 Department of Cardiology, Affiliated Hospital of Nantong University, Medical School, Nantong University, Nantong, Jiangsu, China; 2 School of Life Sciences, Nantong University, Nantong, Jiangsu, China

**Keywords:** cardiac autonomic regulation, intrinsic cardiac nervous system (ICNS), neuro-cardiac interaction, single-cell RNA sequencing, spatial transcriptomics

## Abstract

The intrinsic cardiac nervous system (ICNS), composed of sympathetic, parasympathetic, and sensory neurons embedded within the heart, plays a pivotal role in regulating cardiac electrophysiology and contractility. Functional imbalance within this network represents a central mechanism driving myocardial infarction, heart failure (HF), atrial fibrillation (AF), and hypertension. Despite decades of investigation, the precise pathophysiological basis of ICNS involvement in cardiovascular disease remains poorly understood. Traditional bulk RNA sequencing, which measures averaged gene expression across pooled cells, fails to capture the cellular heterogeneity of ICNS, particularly rare neuronal and glial subpopulations. Consequently, critical regulatory pathways have been obscured. Single-cell RNA sequencing (scRNA-seq) has overcome this limitation by enabling transcriptional profiling at single-cell resolution. This breakthrough has refined cellular taxonomy within the ICNS and revealed dynamic molecular networks that underlie heart-neural interactions. This review summarizes technological breakthroughs in single-cell isolation, lineage tracing, and spatial transcriptomics, and highlights key discoveries including the identification of cardiac nexus glia (CNG), arrhythmogenic neuron subtypes, and neuroimmune interactions in cardiovascular diseases. We also discuss existing methodological bottlenecks and current challenges in data integration, species translation, and clinical application, and future research goals that could bridge the gap between molecular insight and therapeutic application.

## Introduction

**FIGURE 1:**
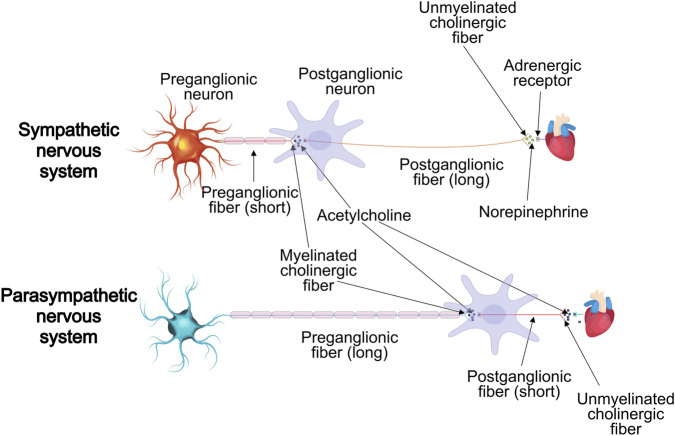
Organization of sympathetic and parasympathetic pathways regulating cardiac function. Sympathetic preganglionic neurons project to postganglionic neurons within the stellate ganglia, which subsequently innervate the heart through long postganglionic fibers and release norepinephrine onto adrenergic receptors. Parasympathetic preganglionic fibers project to intrinsic cardiac ganglia located near the heart, where short postganglionic neurons release acetylcholine to modulate cardiac chronotropic and dromotropic activity. The schematic illustrates the basic anatomical organization of autonomic neuro-cardiac regulation relevant to the intrinsic cardiac nervous system (ICNS). Created with MedPeer (medpeer.cn).

The autonomic nervous system (ANS) serves as a fundamental regulator of internal physiology, maintaining metabolic homeostasis and coordinating visceral responses to environmental and internal stimuli (Gibbons, 2019). Through sympathetic and parasympathetic divisions, the ANS controls cardiac contraction, vascular tone, glandular secretion, and visceral motility (Gordan et al., 2015; Hafez and Chang, 2025). Within this network, the intrinsic cardiac nervous system (ICNS) functions as a localized neural control center embedded in the heart, modulating cardiac rhythm, contractility, and adaptation to stress (Beaumont et al., 2013; Giannino et al., 2024). Sympathetic activation exerts positive chronotropic and inotropic effects on cardiac function, whereas parasympathetic (vagal) signaling provides complementary inhibitory regulation of heart rate and myocardial contractility (Rajendran et al., 2024). Neurons located in the nucleus ambiguus (NA) and dorsal motor nucleus (DMN) of the medulla project via the vagus nerve to the heart, producing negative chronotropic and inotropic effects (Massari et al., 1995) (Figure 1). Animal studies demonstrate that acute myocardial infarction and chronic heart failure are associated with sympathetic hyperactivity and vagal suppression (Jardine et al., 2005; Wu et al., 2023; Zhang and Anderson, 2014). Clinical parameters such as heart-rate variability, baroreflex sensitivity, and plasma catecholamine levels predict morbidity and mortality in myocardial infarction and heart-failure patients (Nolan et al., 1998; Osterziel et al., 1995; Cohn et al., 1984).

Autonomic imbalance also plays a central role in atrial fibrillation (AF). Following the seminal work of Haïssaguerre et al. identifying ectopic discharges from the pulmonary veins (Haïssaguerre et al., 1998), subsequent studies revealed that sympathetic and parasympathetic fibers densely co-localize in pulmonary venous tissue. Their synergistic activation can precipitate arrhythmogenic bursts that initiate AF. Indeed, autonomic dysregulation represents an important contributing component of multiple cardiovascular disorders, including hypertension, myocardial infarction, and heart failure, and is frequently accompanied by neuronal remodeling, altered neurotransmitter expression, and heterogeneous innervation (Giannino et al., 2024; Chakraborty et al., 2023; Elia and Fossati, 2023; Sridharan et al., 2022).

Although dysfunction of ICNS has been implicated in a range of cardiovascular diseases, the cellular diversity and molecular mechanisms underlying ICNS development and pathology remain poorly understood.

Traditional transcriptomic studies have generated valuable insights into gene expression alternation linked to dysfunction of ICNS (Giannino et al., 2024; Kleiger et al., 1987; Chen J. et al., 2021; Menard et al., 2014), but bulk sequencing provides only averaged gene expression profiles across diverse cellular populations, masking rare or functionally specialized cell populations and dynamic transcriptional changes. The advent of single-cell RNA sequencing (scRNA-seq) has revolutionized our ability to dissect cellular heterogeneity, enabling the identification of cellular heterogeneity and novel cell types, lineage relationships, intercellular signaling pathways, and disease-specific transcriptional signatures (Jovic et al., 2022; Pedroni et al., 2024; Yamada and Nomura, 2020). This technological advance has become a powerful tool for elucidating the complex cellular architecture and developmental dynamics of the ICNS. In this review, we organize recent advances in ICNS single-cell and spatial transcriptomics into three interconnected conceptual layers: (1) developmental origin and lineage specification, (2) cellular diversity and neuro-glial-immune organization, and (3) functional neuro-cardiac interactions in health and disease. This framework aims to move beyond descriptive cataloging toward an integrated understanding of how molecular heterogeneity contributes to electrophysiological regulation, adaptive remodeling, and cardiovascular pathology.

### Advances in single-cell sequencing for cardiac autonomic development

This section summarizes how single-cell technologies have enabled reconstruction of developmental trajectories within the ICNS, providing insight into lineage specification and cellular maturation.

### Progress in single-cell isolation and transcriptomic profiling

Single-cell RNA sequencing (scRNA-seq) has redefined how researchers interrogate cardiac and neural development. The first report of single-cell transcriptome sequencing by Tang and colleagues in 2009 employed manual micromanipulation to isolate individual mouse oocytes and blastomeres (Tang et al., 2009). Although labor-intensive and low-throughput, this pioneering work revealed previously unseen transcriptional complexity.

Over subsequent years, cell-isolation platforms evolved dramatically. The development of microfluidic platforms such as Drop-seq and 10x Genomics has enabled simultaneous encapsulation and barcoding of thousands of cells with high efficiency (Klein et al., 2015; Macosko et al., 2015; Zheng et al., 2017). Three major microfluidic strategies are now established for single-cell isolation: integrated fluidic circuits (IFCs), microwell arrays, and droplet systems. IFC devices miniaturize multiple microchannels onto a chip, allowing precise capture of rare cells from complex tissues such as the heart, while minimizing reagent consumption. Microwell approaches partition cells into thousands of small chambers for parallel reactions, reducing population-level averaging. Droplet-based systems, by contrast, encapsulate single cells with barcoded beads in oil emulsions, enabling high-throughput and cost-effective profiling but posing challenges in sample recovery and downstream data handling (Hosic et al., 2016; Han et al., 2018; Hwang et al., 2021). These advances have accelerated cardiac transcriptomic studies. In 2016, two studies applied scRNA-seq to embryonic mouse cardiomyocytes, generating the first single-cell temporal atlas of cardiac gene expression and revealing stage-specific regulatory programs during heart morphogenesis (DeLa et al., 2016; Li et al., 2016). Two years later, Nomura et al. and Gladka et al. independently profiled healthy and diseased hearts, identifying disease-specific fibroblast and cardiomyocyte subpopulations that drive pathological remodeling (Nomura et al., 2018; Gladka et al., 2018). A landmark contribution by Chen and co-workers integrated scRNA-seq with spatial validation (smFISH) to map derivatives of cardiac neural-crest cells (CNCCs) across eight developmental stages from E10.5 to P7 (Chen W. et al., 2021). Analysis of over 34,000 CNCC-derived cells revealed their differentiation into cardiac pericytes and microvascular smooth-muscle cells, establishing CNCCs as a third cellular source of the heart wall in addition to epicardial and endothelial origins. Although this study focused on normal development rather than congenital heart-disease models, it opened new avenues for exploring CNCC-related malformations such as DiGeorge and CHARGE syndromes. The discovery also highlighted the regenerative potential of CNCC-derived lineages for cardiac repair (Chen et al., 2016; Avolio and Madeddu, 2016).

Collectively, these developments underscore that continuous innovation in single-cell isolation and sequencing platforms has substantially expanded our understanding of cardiac neurodevelopment. Each technological generation—from micromanipulation to droplet microfluidics—has enhanced resolution, throughput, and sensitivity, paving the way for detailed mapping of ICNS cell types and their developmental trajectories.

### Lineage-tracing innovations for developmental reconstruction

Traditional lineage-tracing approaches—such as dye injection or fluorescent labeling followed by manual clonal analysis—are limited by low throughput and short labeling duration (Kretzschmar and Watt, 2012; Hsu, 2015). The integration of scRNA-seq with genetic barcoding now allows cell lineages to be reconstructed at single-cell resolution. In these systems, inheritable DNA barcodes are introduced into progenitor cells and read out through high-throughput sequencing, enabling researchers to infer developmental relationships among diverse cell states (Baron and Van Oudenaarden, 2019; McKenna and Gagnon, 2019; Spanjaard et al., 2018).

Most current strategies employ CRISPR–Cas9-based recording of barcodes arranged as tandem repeats under 300 bp in length (Bowling et al., 2020; McKenna et al., 2016). However, practical challenges remain: low efficiency of barcode capture, incomplete editing, and convergent mutations can all confound lineage inference (Li et al., 2023). To address these issues, Chen and colleagues recently developed *DuTracer*, a dual-nuclease lineage-recording platform that simultaneously uses Cas9 and Cas12a systems (Chen et al., 2025). By separating the activity of these orthogonal nucleases, *DuTracer* generates deeper and more diverse lineage trees than single-enzyme systems. Validation in HEK293T cells, mouse embryonic stem cells, embryoid bodies, and neuro-mesodermal organoids demonstrated that neural-mesodermal progenitors exhibit distinct lineage biases toward neural or mesodermal fates. Although *DuTracer* has yet to be tested *in vivo*, it represents a conceptual advance for tracing cardiac-neural lineage relationships. Future adaptations—such as combining CRISPR editing with light-responsive promoters to reduce background leakage (Zhao et al., 2018)—may enable precise temporal control of lineage recording within cardiac neurons and glia. Integrating these approaches with human organoid models could further reveal species-specific aspects of human cardiac-autonomic development.

### Spatial and multimodal platforms enabling cardiac neuro-anatomical mapping

Because spatial context is essential to understanding cell communication, combining scRNA-seq with spatial transcriptomics has become a major frontier. In a landmark study, Asp et al. correlated single-cell data from human embryonic hearts with spatially resolved transcriptomic maps, constructing the first organ-wide atlas of developing human heart gene expression (Asp et al., 2019). Although limited to four embryonic samples and lacking early cardiac-tube and late fetal stages, this work established a foundational framework for future integrative analyses.

Similarly, Li and colleagues employed integrated spatial and single-cell transcriptomics (Stereo-seq plus scRNA-seq) to generate a four-dimensional spatiotemporal atlas of zebrafish heart regeneration (Li et al., 2025). Sampling eight timepoints from injury to full regeneration, they identified regeneration-specific genes (*atp6ap2*, *ifrd1*) and validated dynamic changes across the myocardial landscape. While species differences in regenerative capacity necessitate cautious extrapolation to mammals, the methodological paradigm of combining Stereo-seq with scRNA-seq is directly applicable to studies of the cardiac nervous system.

Standard droplet-based systems (e.g., 10x Chromium) are optimized for small, easily dissociated embryonic or neonatal cardiomyocytes, but large mature neurons are often lost during enzymatic digestion (Marsh et al., 2022; Denisenko et al., 2020; Wang et al., 2021). To overcome this, researchers have adopted single-nucleus RNA sequencing (snRNA-seq), which profiles nuclear RNA and thus circumvents stress responses associated with tissue dissociation. This approach is especially suitable for archived or frozen specimens and large neural cells, although it captures only nuclear transcripts, potentially underrepresenting mature cytoplasmic mRNAs (Lake et al., 2019; Slyper et al., 2020). Using a staged protocol, Ge et al. performed snRNA-seq on the superior cervical and stellate ganglia of adult mice, labeling nuclei with oligonucleotide tags (HTOs) to trace their ganglionic origin (Ge et al., 2022). They successfully captured neuronal, satellite-glial, and endothelial nuclei, providing a valuable resource for studying cardiac sympathetic ganglia at single-cell resolution. Notably, differences between nuclear and cytoplasmic RNA pools may influence interpretation of activity-dependent transcription, and further studies are needed to assess reproducibility across species and ganglia.

In summary, advances in single-cell isolation, lineage tracing, and spatial transcriptomics have jointly revolutionized cardiac autonomic research. These technologies now enable precise characterization of neuronal and glial heterogeneity, reconstruction of developmental trajectories, and mapping of molecular networks within the three-dimensional cardiac environment. Together, they lay the groundwork for dissecting the mechanisms that couple neural development with cardiac physiology and disease.

### Mechanisms of cardiac–neural interactions and disease relevance

Beyond developmental processes, single-cell approaches also reveal how cellular diversity within the ICNS contributes to functional neuro-cardiac communication and disease susceptibility.

### The cardiac–neural axis: from anatomical connectivity to molecular regulation

More than a decade ago, neurologists recognized that cerebral injury can precipitate profound cardiovascular complications (Samuels, 2007). The intrinsic cardiac nervous system (ICNS)—often described as a “little brain” within the heart (Armour, 2008). Modern single-cell and multi-omics approaches have now begun to uncover the molecular basis of this neuro-cardiac interface.

Through the combination of single-cell sequencing, lineage tracing, and advanced imaging, researchers have mapped multiple levels of the cardiac–neural axis, from developmental origins to physiological regulation. For example, using the *DuTracer* platform, Chen and colleagues successfully distinguished the developmental origins of cells from the first and second heart fields and identified *Foxb1* as a transcriptional regulator guiding the neural lineage differentiation of neuro-mesodermal progenitors (Chen et al., 2025). This discovery links early embryonic patterning directly to cardiac neuronal specification, providing a molecular entry point for studying congenital autonomic defects.

### Insights from model organisms: molecular and functional diversity

In a seminal study, Pedroni et al. integrated scRNA-seq, neurochemical labeling, and electrophysiology to characterize the ICNS of adult zebrafish (Pedroni et al., 2024). Immunostaining for HuC/D+ neurons revealed their distribution across all cardiac regions, while single-cell transcriptomic profiling identified 22 distinct cellular clusters comprising cardiomyocytes, neurons, and supporting glial cells. The authors discovered that variations in neuronal firing properties directly correlate with heart rate, and that asynchronous neurotransmitter release by intracardiac neurons disrupts myocardial contractility. Furthermore, they identified “bursting neurons” with electrophysiological signatures associated with arrhythmogenesis, including spontaneous depolarizations and rebound firing after hyperpolarization—phenomena consistent with earlier observations in mammalian models (Smith, 1999). These findings demonstrate that intracardiac neurons exhibit remarkable molecular and functional heterogeneity, with subsets likely specialized for rhythm control, signal integration, or pathophysiological remodeling.

These findings also raise the possibility that transcriptomic heterogeneity may extend beyond individual neuronal subtypes to region-specific organization within the ICNS. Distinct ganglionated plexi, including the right atrial ganglionated plexus (RAGP) and left atrial ganglionated plexus (LAGP), differ in anatomical connectivity and electrophysiological influence, particularly in regulating sinoatrial node activity and atrioventricular conduction (Moss et al., 2021). Although current single-cell datasets remain limited in spatial resolution, integration with anatomical mapping and spatial transcriptomics may enable future identification of ganglion-specific neuronal programs and regional neuro-cardiac regulation.

### Cardiac nexus glia as critical modulators of neural–cardiac crosstalk

Beyond neurons, the discovery of cardiac nexus glia (CNG) has added a new dimension to cardiac neurobiology. Kikel-Coury and colleagues identified glial populations in zebrafish, mice, and humans that express canonical astrocytic markers (Kikel-Coury et al., 2021). ScRNA-seq analyses revealed that these CNGs differentiate under the influence of the secreted factor meteorin, which activates the Jak/Stat3 pathway to drive glial maturation. Functional ablation of CNGs delayed or impaired cardiac axon innervation, especially in the outflow tract, and increased susceptibility to ventricular arrhythmias. These glial cells thus act as critical regulators of neurodevelopmental timing and electrophysiological stability, coordinating sympathetic and parasympathetic inputs to maintain cardiac rhythm homeostasis.

### Neuro–immune–cardiac interactions in disease pathogenesis

Emerging evidence suggests that immune cells serve as intermediaries between the nervous and cardiovascular systems. Using a mouse model of middle cerebral artery occlusion (MCAO), Lin et al. demonstrated that ischemic stroke triggers secondary cardiac injury through monocyte-derived macrophage expansion (Lin et al., 2024). Single-cell RNA sequencing identified a subset of cardiac macrophages expressing CCL2 and activating the NLRP3 inflammasome, driving myocardial inflammation and dysfunction. This neuro–immune communication highlights the systemic reach of autonomic dysregulation and suggests that modulation of inflammatory cascades may ameliorate stroke-associated cardiac syndromes.

Complementary findings in heart failure with preserved ejection fraction (HFpEF) models further underscore the importance of neuro–immune pathways. In a recent *Circulation Research* report, Venkatesan et al. showed that transcutaneous vagus nerve stimulation (tVNS) improved cardiac function by reprogramming resident macrophage subsets (Venkatesan et al., 2025). Single-cell RNA-seq and flow cytometry revealed that tVNS reduced proinflammatory CCR2+ macrophages expressing Spp1 while inducing reparative TLF+/MHC-II+ subsets expressing Igf1. These observations provide mechanistic evidence that vagal modulation exerts cardioprotective effects via immune rebalancing, offering a potential therapeutic strategy for HFpEF and other neurogenic cardiac disorders.

Current evidence suggests that autonomic signaling can influence inflammatory remodeling and immune-cell activation within the heart (Lin et al., 2024; Venkatesan et al., 2025); however, the precise neuronal and glial subtypes responsible for orchestrating these interactions remain incompletely resolved. Most existing studies identify broad associations between autonomic dysregulation and immune activation rather than subtype-specific causal mechanisms within the ICNS (Lin et al., 2024; Venkatesan et al., 2025). Further integration of spatial transcriptomics and functional perturbation approaches will likely be required to define the cellular specificity and bidirectional dynamics of neuro–immune communication in cardiovascular disease (Longo et al., 2021).

Together, these studies converge on a paradigm in which the heart and its intrinsic neural network operate as a bidirectional, multi-layered communication system. Neurons orchestrate electrical and contractile behavior through neurotransmitter release and synaptic modulation, while glial and immune cells dynamically shape this environment by regulating inflammation, trophic signaling, and metabolic support. The integration of single-cell sequencing with electrophysiology and spatial mapping is now revealing how these elements cooperate to maintain physiological stability—and how their disruption precipitates disease.

In summary, the cardiac–neural interface functions not merely as a regulatory relay but as an adaptive microcircuit whose plasticity underlies both cardiac resilience and vulnerability. Single-cell and spatial transcriptomic technologies have provided the molecular tools to dissect this complexity, paving the way toward targeted manipulation of neuro-cardiac pathways in cardiovascular disease.

## Future directions and clinical translation

### Technical bottlenecks and emerging solutions

The rapid evolution of single-cell sequencing has profoundly reshaped our understanding of the ICNS, but several key challenges continue to limit its broader application. Chief among these is the difficulty of preserving cellular integrity during tissue dissociation. Adult cardiac tissue is dense, fibrous, and rich in extracellular matrix (ECM), which makes enzymatic dissociation particularly harsh on delicate neurons and glial cells; long incubations with collagenases/proteases and mechanical trituration tend to fragment processes, reduce viability, and selectively deplete large or morphologically complex cell types. Enzymatic and mechanical stress can also provoke immediate-early and stress-response transcriptional programs, producing dissociation-induced artefacts that distort the *in vivo* transcriptome and bias cell-type composition (Litviňuková et al., 2020; Mattei et al., 2020; Gladka, 2021).

Single-nucleus RNA-seq (snRNA-seq) enables the profiling of archived or frozen cardiac and neural tissues by sequencing nuclear RNA, thereby avoiding cell-isolation biases and improving recovery of fragile cell types (Lei et al., 2021; Bakken et al., 2018). However, because it samples predominantly nascent and intron-containing nuclear transcripts, snRNA-seq can underrepresent mature cytoplasmic mRNAs and some post-transcriptionally regulated messages involved in rapid neuronal signaling (Lacar et al., 2016; Lake et al., 2017; Hu et al., 2017). Future methodological improvements should therefore aim to integrate scRNA-seq and snRNA-seq datasets, or to develop hybrid protocols that preserve cytoplasmic content while maintaining sample viability.

Another pressing challenge is cross-species extrapolation. Much of our current knowledge derives from zebrafish and mouse models, which differ substantially from humans in cardiac structure, innervation density, and regenerative potential. Human single-cell and spatial transcriptomic studies of the developing heart have expanded rapidly in recent years, providing increasingly detailed insight into human cardiac cellular organization and developmental patterning (Asp et al., 2019; Lázár et al., 2025). However, specifically resolved datasets focusing on the human ICNS remain relatively limited compared with those available in experimental animal systems. This limitation largely reflects challenges in tissue procurement, anatomical accessibility, and preservation of fragile neural structures during tissue processing.

Current human cardiac single-cell and single-nucleus datasets predominantly derive from atrial appendage tissue, ventricular myocardium, surgical resections, donor hearts, or end-stage heart-failure specimens. Most studies primarily resolve cardiomyocytes, fibroblasts, endothelial cells, immune cells, and vascular-associated stromal populations, whereas neuronal and glial populations are typically represented at substantially lower abundance and remain incompletely characterized in current human ICNS-focused datasets (Litviňuková et al., 2020; Simonson et al., 2023). The low abundance and anatomical dispersion of intrinsic cardiac neurons, together with technical challenges in preserving fragile neural structures during tissue dissociation and nuclei isolation, continue to limit comprehensive profiling of human ICNS neuronal diversity. Consequently, many translational interpretations currently remain hypothesis-generating and require further validation using spatially resolved and functionally integrated human datasets.

At present, the principal translational value of human ICNS-related single-cell datasets lies in identifying conserved cellular programs, candidate neuro-cardiac signaling pathways, and disease-associated cellular states that may guide future mechanistic investigation and targeted validation. However, limited neuronal representation, restricted tissue accessibility, and the predominance of end-stage disease specimens continue to constrain direct clinical extrapolation and therapeutic implementation. Therefore, current translational applications should be interpreted primarily as an emerging framework for hypothesis generation rather than as immediately actionable precision-medicine strategies.

Nevertheless, increasing evidence from large-animal and human studies has begun to complement findings derived from zebrafish and rodent systems (Arora et al., 2003; Vaseghi and Shivkumar, 2008). Investigations in porcine, ovine, and human cardiac tissues have provided critical insight into ganglionated plexus organization, autonomic remodeling, and translational electrophysiology (Moss et al., 2021; Arora et al., 2003; Tompkins et al., 2025). Pioneering work by groups including Kalyanam Shivkumar and Rajanikanth Vadigepalli has contributed substantially to defining ICNS architecture and functional connectivity in clinically relevant contexts (Moss et al., 2021; Vaseghi and Shivkumar, 2008; Zhu et al., 2019). These comparative studies highlight the importance of integrating datasets across species to improve translational relevance and bridge experimental findings with human cardiovascular physiology.

Important biological differences between species further complicate direct translation. The zebrafish heart exhibits robust regenerative capacity—e.g., resected ventricles fully regenerate via proliferation of pre-existing cardiomyocytes—a capability that adult mammals largely lack, although a transient regenerative window exists in neonatal mice (Poss et al., 2002). Although the overall anatomy of the intrinsic cardiac nervous system (ICNS) in mice resembles that in larger mammals, detailed electrophysiological recordings and single-cell/molecular profiling reveal species-specific differences in intrinsic excitability (tonic vs. phasic firing), ion-channel expression, and neurochemical signatures—for example, mouse intracardiac cholinergic neurons commonly co-express noradrenergic enzymes and diverse neuropeptides. These differences have important implications when extrapolating murine ICNS physiology to human cardiac autonomic regulation or when designing translational interventions (Lizot et al., 2022). To help bridge this gap, development of human induced pluripotent stem cell (hiPSC)-derived cardiac–neural co-culture systems and 3D cardiac organoids may provide valuable platforms for modeling human-specific neuro-cardiac development and disease mechanisms.

Computationally, one of the major challenges lies in integrating massive, multimodal datasets (Longo et al., 2021; Hao et al., 2021; Chen et al., 2024). ScRNA-seq, snRNA-seq, and spatial transcriptomics routinely generate noisy, sparse, and high-dimensional measurements spanning millions of cells, developmental stages, and anatomical regions. This scale and heterogeneity place substantial demands on memory, computational resources, and downstream analytical frameworks. Conventional clustering and dimensional-reduction methods may oversimplify biological continua by forcing discretized partitions and overlooking gradual, context-dependent cell-state transitions.

To address these limitations, artificial intelligence (AI) and machine learning (ML) approaches are increasingly being incorporated into single-cell analysis pipelines. Graph-based neural networks, generative models, and multimodal integration frameworks provide new opportunities to reconstruct complex neuro-cardiac interactions across developmental and pathological states (Ge et al., 2025). In addition, trajectory inference algorithms can model lineage progression among neuronal and glial populations, while cell–cell communication analyses infer signaling relationships between neural, immune, and stromal compartments. Integration of transcriptomic, spatial, and electrophysiological information further enables reconstruction of region-specific neuro-cardiac networks. Emerging multimodal strategies integrating transcriptomic and chromatin-accessibility datasets, including single-cell ATAC-seq–based analyses, may further improve understanding of regulatory programs underlying ICNS development and disease-associated remodeling (Hao et al., 2021; Ma et al., 2020). Collectively, these computational advances offer a critical bridge between large-scale molecular profiling and systems-level understanding of ICNS organization and function. Importantly, computationally defined clusters, trajectories, and inferred cellular states do not necessarily correspond to stable biological entities or functionally distinct populations. Dissociation-associated artifacts, sequencing depth, batch effects, and analytical assumptions may all influence transcriptomic structure and trajectory inference. Therefore, orthogonal validation approaches—including electrophysiology, lineage tracing, spatial mapping, and functional perturbation experiments—remain essential for confirming the biological relevance of single-cell findings.

Finally, despite reductions in per-cell cost, high-depth sequencing and multimodal analyses remain financially demanding, restricting sample size and replication. Recently, the STAMP (Single-cell Transcriptomics Analysis and Multimodal Profiling) technique has emerged as a cost-efficient alternative that bypasses fluorescence-activated cell sorting bias and is especially suitable for irregularly shaped neurons (Pitino et al., 2025). Adoption of such scalable, low-cost technologies will democratize access to single-cell research and accelerate translation toward clinical contexts.

### Clinical implications and translational prospects

Single-cell and spatial transcriptomic studies are already beginning to influence translational cardiovascular research. For instance, scRNA-seq of human aortic valve tissue identified upregulation of lumican (LUM) in calcific aortic valve disease (CAVD); genetic deletion of *Lum* in mice significantly reduced valvular calcification (Huang et al., 2024). This finding exemplifies how high-resolution transcriptomic mapping can reveal novel therapeutic targets within cardiovascular tissues. Similarly, Qi and colleagues used *in vivo* adenine base editing (AAV9-ABEmax) to correct pathogenic *Scn5a* mutations in a murine model of long QT syndrome type 3, effectively normalizing cardiac rhythm (Qi et al., 2024). Together, these studies illustrate how molecular insights from single-cell data can guide precise genetic or pharmacological interventions.

Looking ahead, two major translational pathways are emerging. First, gene-targeted therapies that leverage knowledge of cell-type–specific expression could enable precise modulation of arrhythmogenic neurons or glial regulators of excitability (Bode et al., 2021). For example, targeted manipulation of CNG-related pathways such as Metrn–Jak/Stat3 may offer a means to stabilize neuro-cardiac communication in rhythm disorders (Kikel-Coury et al., 2021; Lee et al., 2015). Second, biomarker discovery based on single-cell signatures could facilitate early detection of autonomic dysfunction before overt cardiovascular disease develops. Expression profiles of stress-responsive neuronal subsets, inflammatory macrophages, or maladaptive fibroblast–glia interactions may serve as predictive markers for heart failure, AF, or post-stroke cardiac complications (Suzuki et al., 2024; Chen et al., 2020). In addition to disease association, single-cell–derived biomarkers may provide insight into early-stage autonomic remodeling before structural or electrophysiological dysfunction becomes clinically detectable. Cell-type–specific transcriptional signatures from neuronal, glial, immune, or stromal populations may help identify latent neuro-cardiac dysregulation and stratify disease susceptibility. Integration with spatial transcriptomics may further improve biomarker specificity by preserving anatomical context within ICNS ganglia and surrounding cardiac tissue. Representative ICNS-related single-cell and spatial transcriptomic studies across species are summarized in Table 1.

**TABLE 1:** Representative single-cell and spatial transcriptomic studies investigating the intrinsic cardiac nervous system (ICNS) across species.

Species	Tissue/Region/Celltype	Technology	Main findings	Ref.
Zebrafish	Adult intracardiac nervous system	scRNA-seq	Identified 22 cellular clusters within the ICNS and revealed electrophysiologically distinct neuronal subtypes associated with arrhythmogenic activity and cardiac rhythm regulation	Pedroni et al. (2024)
Zebrafish	Regenerating heart	Stereo-seq + scRNA-seq	Constructed a 4D spatiotemporal atlas of heart regeneration and identified regeneration-associated molecular programs	Li et al. (2025)
Mouse	Cardiac neural crest derivatives during development	scRNA-seq + spatial validation (smFISH)	Constructed a developmental atlas of cardiac neural crest cell derivatives and identified differentiation trajectories toward pericytes and vascular smooth-muscle cells	Chen et al. (2021b)
Mouse	Cardiac macrophage following ischemic stroke	scRNA-seq	Identified inflammatory macrophage subsets associated with stroke-induced cardiac dysfunction and neuro–immune remodeling	Lin et al. (2024)
Mouse	Heart tissue in HFpEF model	scRNA-seq	Demonstrated that vagus nerve stimulation alters cardiac macrophage composition and improves cardiac function through immune reprogramming	Venkatesan et al. (2025)
Pig	Right atrial ganglionic plexus	scRNA-seq	Mapped paracrine signaling networks within the ICNS and highlighted cellular communication pathways relevant to neuro-cardiac regulation	Moss et al. (2021)
Human	Developing human heart	Spatial transcriptomics + scRNA-seq	Generated the first organ-wide spatiotemporal atlas of human heart development with spatially resolved gene-expression mapping	Asp et al. (2019)
Human	Developing human heart	scRNA-seq + Spatial transcriptomics	Constructed a high-resolution spatiotemporal atlas of the developing human heart, identifying 72 fine-grained cell states and mapping emerging autonomic innervation, cardiac conduction-system organization, and spatial neuro-cardiac niches	Lázár et al. (2025)
Zebrafish/Mouse/Human	Cardiac glial populations	scRNA-seq	Identified cardiac nexus glia (CNG) and demonstrated their role in regulating both the sympathetic and parasympathetic system	Kikel-Coury et al. (2021)

At a broader level, a structured translational workflow may help bridge molecular discovery and clinical implementation. Such a framework may include four sequential stages: (1) identification of disease-associated cellular states through single-cell transcriptomic profiling; (2) prioritization of candidate molecular targets using spatial validation and multimodal integration; (3) development of targeted interventions, including neuromodulation, pharmacological modulation, or gene editing strategies; and (4) clinical stratification based on predictive biomarkers and patient-specific molecular signatures. This stepwise strategy may facilitate the systematic translation of ICNS-related molecular discoveries into precision cardiovascular medicine. Nevertheless, many of these translational applications remain at an early exploratory stage, particularly in the context of the human ICNS, where limited access to healthy tissue and difficulties in longitudinal sampling continue to constrain mechanistic validation and clinical implementation.

Finally, integration of single-cell multi-omics with *in vivo* imaging, electrophysiological monitoring, and AI-driven analytics will further refine the diagnosis and treatment of autonomic cardiovascular diseases. Such efforts could ultimately transform mechanistic understanding into precision medicine strategies that target neuro-cardiac pathways at the earliest stages of dysfunction.

## Concluding remarks

Single-cell sequencing has redefined the conceptual landscape of cardiac neurobiology. By providing cellular and molecular resolution of the intrinsic cardiac nervous system, it has illuminated the developmental origins, lineage relationships, and functional diversity of cardiac neurons and glia. These insights reveal that the heart’s “little brain” is not merely a passive relay of autonomic commands but an active and adaptive network that shapes cardiovascular health and disease.

As new technologies integrate transcriptomic, spatial, and functional dimensions, future research will move beyond cataloging cell types to decoding dynamic neuro-cardiac interactions in real time. The ultimate goal is to harness this knowledge for therapeutic innovation—repairing or reprogramming neural circuits to restore cardiac homeostasis. The convergence of single-cell biology, genetic engineering, and computational modeling thus heralds a new era of neuro-cardiac precision medicine.
